# Reply to R. Freitas-Junior et al

**DOI:** 10.1200/GO.23.00147

**Published:** 2023-07-10

**Authors:** Cristiano A.A. Resende, Rodrigo Dienstmann, Max S. Mano

**Affiliations:** Cristiano A.A. Resende, MD, Oncoclínicas, Brasília, Brazil; and Rodrigo Dienstmann, MD and Max S. Mano, MD, Oncoclínicas, São Paulo, Brazil

First, we would like to thank the authors of this letter^1^ for referring to our manuscript published in November 2022 in *JCO Global Oncology*, in which we addressed the impact of the COVID-19 pandemic on staging at diagnosis of patients with breast cancer in a private service in Brazil.^2^ Freitas-Junior et al recently published a similar analysis in the journal *International Journal of Public Health* but focused on patients from the Brazilian public health system and older than 50 years.^3^

We are facing an apparently unsolvable situation in the country which is the growing social and economic inequality—a problem that has also affected many other countries.^4^ As mentioned by Freitas-Junior et al, Brazil has historically been plagued by unequal access to screening tests for breast cancer—especially among women without private health insurance coverage^5^ who, even in the prepandemic period, were more likely to be diagnosed at advanced stages.^6^ The data reported in both studies are coherent in terms of showing that, after the onset of the COVID-19 pandemic, there has been a significant increase in cases diagnosed in advanced stages (defined as stages III and IV) compared with the prepandemic period.^2,3^

The informal comparison between the two studies proposed by Freitas-Junior et al suggests that the impact of the COVID-19 pandemic was more devastating in the public than in the private sector. Although we agree that this statement potentially reflects the reality, it is only an inference; it cannot be proved because the studies used different databases and methodologies (including, for instance, how events were documented and how outcomes were measured).

As reported at the conclusion of our article, we believe that the short-term consequences of the pandemic are already apparent, and they could be greater in low- and middle-income countries. The potential long-term impact of the COVID-19 pandemic on disease staging at diagnosis will remain an important question for years to come; we take the opportunity to highlight the continued need for interventions aiming at promoting equity of access to health care in Brazil—including screening tests and effective treatments.
